# Automated, Computer Generated Reminders and Increased Detection of Gonorrhoea, Chlamydia and Syphilis in Men Who Have Sex with Men

**DOI:** 10.1371/journal.pone.0061972

**Published:** 2013-04-17

**Authors:** Huachun Zou, Christopher K. Fairley, Rebecca Guy, Jade Bilardi, Catriona S. Bradshaw, Suzanne M. Garland, Jun Kit Sze, Afrizal Afrizal, Marcus Y. Chen

**Affiliations:** 1 Sexual Health Unit, School of Population Health, University of Melbourne, Melbourne, Australia; 2 Melbourne Sexual Health Centre, Alfred Health, Melbourne, Australia; 3 Kirby Institute, University of New South Wales, Sydney, Australia; 4 Department of Epidemiology and Preventative Medicine, Monash University, Melbourne, Australia; 5 Department of Microbiology and Infectious Diseases, Royal Women’s Hospital, Melbourne, Australia; 6 Department of Obstetrics and Gynaecology, University of Melbourne, Melbourne, Australia; 7 Murdoch Childrens Research Institute, Melbourne, Australia; University of Toronto, Canada

## Abstract

**Background:**

Guidelines recommend frequent screening of men who have sex with men (MSM) for sexually transmissible infections (STIs) but few interventions have demonstrated increased testing and detection of bacterial STIs among MSM in controlled studies.

**Methods:**

We used automated text message and email reminders generated by computer assisted self-interview (CASI) to remind MSM to retest for syphilis. We compared clinic visits, STI testing and detection rates over 12 month between men receiving reminders (reminder group) and men not offered the reminders (concurrent control group).

**Results:**

Men who chose 3-monthly reminders had more clinic visits (median 3 vs 1) and higher testing rates for pharyngeal gonorrhoea (67.0% vs 33.6%), rectal gonorrhoea (62.7% vs 31.1%), urethral chlamydia (67.3% vs 39.3%), rectal chlamydia (62.9% vs 31.3%), syphilis (67.0% vs 39.3%) and HIV (64.9% vs 36.7%) (all *p*<0.001) than concurrent controls, within 12 months after their first visit. Also, men receiving reminders had a higher combined testing rate for all the aforementioned STIs at a same visit (55.7% vs 25.5%, *p*<0.001) compared with concurrent controls. This association remained after adjusting for differences in characteristics between the two groups (adjusted odds ratio:1.77, 95% confidence interval:1.51-2.08). Men receiving reminders also had a higher detection rate of: rectal gonorrhoea (3.7% vs 1.2%, *p* = 0.001), urethral chlamydia (3.1% vs 1.4%, *p* = 0.027), rectal chlamydia (6.6% vs 2.8%, *p*<0.001), and early, latent syphilis (1.7% vs 0.4%, *p* = 0.008) compared with concurrent controls.

**Conclusion:**

This is the first study to demonstate that a fully automated reminder system using CASI was associated with increased detection of bacterial STIs among MSM.

## Introduction

Internationally, high prevalence rates of bacterial sexually transmissible infections (STIs) such as gonorrhoea, chlamydia and syphilis have been seen among men who have sex with men (MSM) [1]–[4]. Furthermore, MSM constitute an important risk group for HIV in many countries [5]–[7] Bacterial STIs are important in this population because of the morbidity they cause and also because they enhance the transmission of HIV. Studies have demonstrated associations between bacterial STIs and HIV seroconversion in MSM [8], [9]. These bacterial STIs are predominantly asymptomatic and therefore require screening for detection [10], [11].

Guidelines, including those from the United States Centers for Disease Control and Prevention recommend at least annual screening of MSM for: pharyngeal and rectal gonorrhoea, urethral and rectal chlamydia, syphilis, and HIV, with 3-6-monthly screening of higher risk men [12,13. In practice, however, rates of testing for these infections among MSM have often been less than optimal [14]–[16].

A systematic review revealed that there have been few controlled studies that have demonstrated the efficacy of specific clinic-based interventions in increasing testing rates for bacterial STIs among MSM, and none have demonstrated increased detection of these infections as a result of improved screening [17]. More frequent screening of MSM for syphilis has been shown to significantly increase the detection of asymptomatic, early syphilis and to reduce the likely duration of infectiousness [18], [19]. Mathematical modelling suggests that more frequent screening of MSM for syphilis would result in a decline in syphilis prevalence particularly if this was targeted to higher risk MSM [20]. These models informed the development of the Australian Syphilis in Gay Men Action Plan which advocated more frequent syphilis testing of MSM as a key strategy for improving syphilis control [21].

In 2009, the Melbourne Sexual Health Centre implemented a fully automated system designed to remind MSM to screen for syphilis at regular intervals, with the expectation that men would return for testing for other bacterial STIs and HIV as well. This system combined computer-assisted self-interview (CASI) to identify MSM and offer reminders, with the subsequent dispatch of reminders for STI screening using text message reminders sent to mobile telephones or emails. The aim of this study was to determine if use of this reminder system was associated with increased frequency of testing for bacterial STIs and, more importantly, increased detection of these infections.

## Methods

### Setting

This study was conducted at the Melbourne Sexual Health Centre, the major public STI clinic in Victoria, Australia.

### Reminder system

As part of their routine clinical care, clients attending the clinic were required to complete a series of questions regarding their sexual history using CASI in the client waiting area before being seen by a clinician. This included questions on the sex of partners, partner numbers and condom use [22]. In February 2009, the clinic implemented an automated system to remind MSM to return regularly to the clinic for syphilis testing. All men who used the CASI and who entered the sex of their partner as being male were shown the following message on the computer screen: “*There is an epidemic of syphilis in gay men. It is spread by both oral and anal sex and often has no symptoms. Your best protection is a blood test every 3 months. We can send you a discreet STI check-up reminder by email or text message.*” Men who chose to receive these reminders were then asked via the computer screen to select how frequently they wished to receive the reminders: every 3 months, 6 months or 12 months, and whether they preferred messages via text message, email or both. Men were then sent the following message at the chosen frequency: “*Your next check-up is now due. Phone for appointment or walk in*”. The entire system – from commencing CASI to the regular dispatch of the reminders – was entirely automated, requiring no human input. Men who declined the service initially were asked again if they wanted to opt in the next time they attended the clinic and undertook CASI.

### Study design

We evaluated the impact of the reminder system using a controlled observation design. We compared the number of clinic visits, STI testing rates and detection of STIs among MSM who chose to have reminders with men who were not offered the reminder service. Men in all comparison groups were individually observed for 12 months after their first visit to determine rates of testing and detection of infections.

### Group definitions

**Figure 1:**
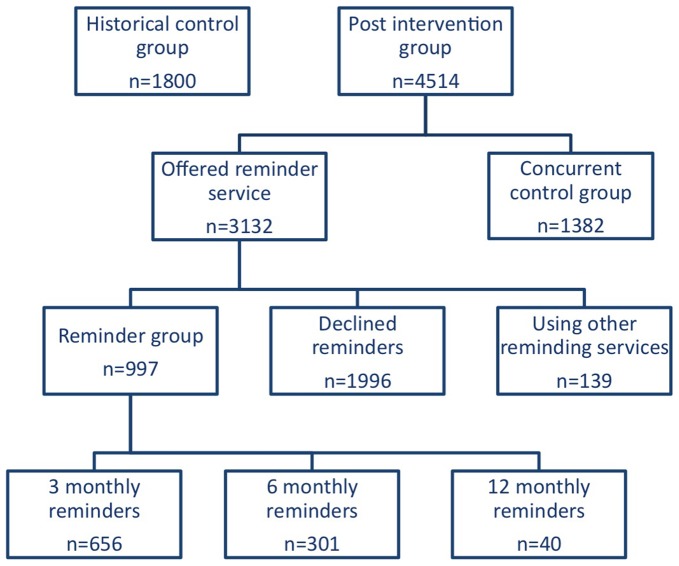
Flow chart of study groups.

We categorize the study population into a number of comparison groups (Figure 1). Men in the **post intervention group** were MSM who attended the clinic after the reminders were implemented, between February 2009 and August 2010. Observation for the last men in this group was completed in August 2011. Within the post intervention group were men who were offered the reminder service (**offered reminder group**) and those who were not offered the reminder service.

Men in the **reminder group** consisted of MSM who attended after the reminders were implemented – between February 2009 and August 2010 – and who were offered and accepted 3, 6 or 12 monthly reminders.

Men in the **concurrent control group** were MSM who attended after the reminders were implemented - between February 2009 and August 2010 – and who were not offered the reminders either because they attended between January and May 2010, when CASI was not operating because the computers had been stolen [22] or because they attended the clinic during certain hours where CASI was not required.

MSM who attended the clinic before the reminder system was implemented - between July 2006 and February 2008 were included in a **historic control group**. Observation for the last men in this group was completed in February 2009.

### Data analysis

Firstly, we compared the risk behaviors, clinic visits, STI testing, and STI detection between men who were offered and men who accepted the reminders and men in the concurrent control group who were not offered the reminders. Secondly, to see if there was an overall effect of the reminders on the clinic population as a whole, we compared clinic visits, STI testing, and STI detection between men in the historic control group and men in the post intervention group.

A Chi-square test was used to compare categorical variables including proportion of men testing for each infection and the proportion found to be infected during the 12 months’ observation period. The Mann-Whitney test was used to compare medians. Univariate logistic and multivariate logistic regressions were used to calculate the crude and adjusted odds ratios for factors associated with complete testing. Stata 19.0 was used to undertake analyses.

### Testing protocols

MSM who attended the clinic were screened according to guidelines [12,13: gonorrhoea testing of the pharynx and rectum by culture, and chlamydia testing of first void urine and a rectal swab by strand displacement assay (Becton Dickinson ProbeTec ET, NJ, USA). These specimens were processed by the Microbiology Diagnostic Unit at the University of Melbourne. In addition, men were offered serology for syphilis using enzyme immunoessay (EIA) and rapid plasma reagin (RPR) together with EIA for HIV. These specimens were processed by the Victorian Infectious Diseases Reference Laboratory.

### Data extraction

Data were extracted from the clinic’s computerized medical records system including: date of clinic visits; date the reminder service was offered; frequency of reminders chosen; number of recent male sexual partners and condom use with male partners; and testing and detection of pharyngeal gonorrhoea, rectal gonorrhoea, urethral chlamydia, rectal chlamydia, syphilis and HIV at each visit.

### Ethics statement

Ethical approval was obtained from the Alfred Hospital Research Ethics Committee on September 26, 2011, project number 373/11. Neither written nor verbal informed consent were obtained from participants, as this project only retrieved existing data from clinical databases in the Melbourne Sexual Health Centre, and data were analyzed anonymously. The Ethics Committee waived the need for written informed consent from the participants. This project did not involve any randomisation, placebo control or withholding/substitution of treatment, programs or services. No intervention was performed on or samples or questionnaires taken from participants.

## Results

### Uptake of the reminders

After the reminders were implemented, 4514 MSM attended the clinic of whom 3132 (69.4%) were offered the reminder service and 1382 (30.6%) were not offered it. Men in the latter group made up the concurrent control group. Among those offered reminders, 997 (31.8%) accepted the reminders, 139 (4.4%) had already registered with another reminder service elsewhere, and 1996 (63.7%) declined the service (Figure 1). Among the 997 men who accepted the service, 656 (65.8%) chose 3-monthly reminders, 301 (30.2%) chose 6-monthly reminders and 40 (4.0%) chose 12 monthly reminders. Given the small uptake of 12 month reminders this group was not further analyzed. A total of 1800 MSM attended the clinic during the preintervention period and were included in the historic control group (Figure 1).

Among men who chose 3-monthly reminders, 340 (51.8%) chose text messages only, 287 (43.8%) chose emails only, and 29 (4.4%) chose to receive both. Among men who chose 6-monthly reminders, 151 (50.2%) chose text messages only, 143 (47.5%) chose emails only, and 7 (2.3%) chose both.

### Characteristics and sexual behaviors

The characteristics of men who were offered reminders (offered reminder group), those who received reminders (reminder group) and those who were not offered reminders (concurrent control group) are shown in Table 1. Compared to men not offered reminders, men who chose 3-monthly reminders were younger (*p*<0.001); had a higher number of male sexual partners (*p*<0.001); and reported greater condom use with partners (p<0.001).

**Table 1:** Characteristics of men in the reminders group compared to the concurrent control group.

	3-monthly reminders			6-monthly reminders			Concurrent controls	
	n	%	*P^a^*	n	%	*P^a^*	n	%
**No. of men**	656			301			1382	
**Age**			<0.001			0.862		
≤30 years	408	62.2		141	46.8		655	47.4
>30 years	248	37.8		160	53.2		727	52.6
**No. of male partners in prior**			<0.001			0.909		
**3 months**								
0–2	241	40.4		142	51.3		564	51.6
≥3	355	59.6		135	48.7		528	48.4
**Condom use with male**			<0.001			0.009		
**partners in prior 3 months**								
Never	41	7.3		26	10.4		162	17.2
Ever	523	92.7		224	89.6		779	82.8
**No. of male partners in prior**			<0.001			0.258		
**12 months**								
0–4	214	35.4		122	43.4		527	47.2
≥5	391	64.6		159	56.6		590	52.8
**Condom use with male**			<0.001			0.035		
**partners in prior 12 months**								
Never	26	4.5		19	7.2		116	11.7
Ever	558	94.5		245	92.8		874	88.3
**Injecting drug use**			0.549			0.284		
Never	591	97.2		280	97.9		1196	96.7
Ever	17	2.8		6	2.1		41	3.3

Notes:

a. P values were calculated on the difference in proportions in men receiving 3-monthly reminders and men not offered reminders, and between men receiving 6-monthly reminders and men not offered reminders.

Compared to men who received reminders, men who declined the reminder service were older, had less sexual partners in the past 3 months and had less condom use (data not shown).

Men in the post-intervention group who were not offered reminders (concurrent control group) were similar to men in the historic control group, with no significant differences with regards to number of male sexual partners and condom use (data not shown).

### Clinic visit rates among men in the reminder group compared to men in the concurrent control group

The proportion of men who returned to the clinic at least once during the 12 month observation period was significantly higher among men receiving 3-monthly reminders (89.5%, *p*<0.001) and 6-monthly reminders (87.7%, *p*<0.001) compared with men in the concurrent control group (70.8%) (Table 2). Those receiving 3-monthly reminders attended a median of 3 times (*p*<0.001) compared with 2 for those receiving 6-monthly reminders (*p* = 0.001), which was significantly higher than the one visit among men not offered reminders. Compared to men in the concurrent control group, men in the offered reminder group had significantly more clinic visits during the 12 month observation period (median number of visit 2 vs 1, *p*<0.001) (Table 2).

**Table 2:** Clinic visits, STI testing and STI detection among men who were offered and received reminders group compared with men in the concurrent control group.

	Offered reminders (n = 3132)			Reminder group									Concurrent control group (n = 1382)	
				3 monthly reminders (n = 656)			6 monthly reminders (n = 301)			3, 6 or 12 monthly reminders (n = 997)				
	n	%	*P^a^*	n	%	*P^a^*	n	%	*P^a^*	n	%	*P^a^*	n	%
% men who had subsequent	2564	81.9	<0.001	587	89.5	<0.001	264	87.7	<0.001	885	88.8	<0.001	978	70.8
visit(s)^b^														
No. of subsequent clinic visits	2(1–36)		<0.001	3(1–36)		<0.001	2(1–14)		0.001	3(1–36)		<0.001	1(1–16)	
(median, range)														
% of men tested at least once														
for each STI at subsequent														
visits:														
Pharyngeal gonorrhoea	1287	50.2	<0.001	393	67.0	<0.001	126	47.7	<0.001	530	59.9	<0.001	329	33.6
Rectal gonorrhoea	1182	46.1	<0.001	368	62.7	<0.001	120	45.5	<0.001	396	44.8	<0.001	304	31.1
Urethral chlamydia	1350	52.6	<0.001	395	67.3	<0.001	130	49.2	0.003	536	60.6	<0.001	384	39.3
Rectal chlamydia	1188	46.3	<0.001	369	62.9	<0.001	119	45.1	<0.001	496	56.1	<0.001	306	31.3
Syphilis	1356	52.9	<0.001	393	67.0	<0.001	137	51.9	<0.001	545	61.6	<0.001	384	39.3
HIV	1261	49.2	<0.001	381	64.9	<0.001	120	45.5	0.010	512	57.8	<0.001	359	36.7
Complete testing^c^	1001	39.0	<0.001	327	55.7	<0.001	102	38.6	<0.001	435	49.2	<0.001	249	25.5
No. (%) of men diagnosed with														
an infection at least once at														
subsequent visits														
Pharyngeal gonorrhoea	36	1.4	0.866	13	2.2	0.185	3	1.1	0.805	16	1.8	0.412	13	1.3
Rectal gonorrhoea	49	1.9	0.162	22	3.7	0.001	2	0.8	0.521	24	2.7	0.023	12	1.2
Urethral chlamydia	62	2.4	0.070	18	3.1	0.027	8	3.0	0.081	26	2.9	0.028	14	1.4
Rectal chlamydia	89	3.5	0.289	39	6.6	<0.001	11	4.2	0.239	51	5.8	0.002	27	2.8
Early syphilis	48	1.9	0.060	19	3.2	0.025	5	1.9	0.680	25	2.8	0.060	15	1.5
Early latent syphilis	27	1.05	0.010	10	1.7	0.008	2	0.8	0.469	12	1.4	0.028	4	0.4
HIV	10	0.4	0.715	5	0.9	0.145	2	0.8	0.305	7	0.8	0.155	3	0.3
Proportion of all tests positive														
in subsequent visits														
Pharyngeal gonorrhoea	38	1.8	0.497	13	1.8	0.533	4	2.2	0.908	17	1.9	0.561	13	2.3
Rectal gonorrhoea	53	2.9	0.477	25	3.9	0.143	2	1.2	0.371	27	3.3	0.332	12	2.3
Urethral chlamydia	67	3.2	0.181	21	2.9	0.430	9	4.5	0.084	30	3.2	0.257	14	2.2
Rectal chlamydia	102	5.5	0.862	47	7.3	0.398	11	6.7	0.772	59	7.2	0.436	31	6.0
Early syphilis	55	2.5	0.877	22	3.0	0.530	5	2.5	0.982	28	3.0	0.568	15	2.5
HIV	10	0.5	0.911	5	0.8	0.658	2	1.1	0.410	7	0.8	0.554	3	0.6
% men with concurrent	27	1.1	0.224	9	1.6	0.071	4	1.5	0.146	13	1.5	0.066	6	0.6
infections at subsequent visits^d^														
% men with repeat infections	19	0.7	0.937	15	2.6	0.003	2	0.8	0.943	17	1.9	0.021	7	0.7
at subsequent visits ^e^														

Notes:

a. P value for the difference between the offered reminder group, 3-monthly and 6-monthly reminder groups and men not offered reminders.

b. All subsequent visits were within 12 months of the first visit after the SMS reminder project commenced.

c. Complete testing refers to testing for: pharyngeal gonorrhoea, rectal gonorrhoea, urethral chlamydia and rectal chlamydia and serology for both HIV and syphilis at the same visit.

d. If a man had two or more of the following STIs detected at the same testing episode in subsequent visits in the12 month observation period: pharyngeal gonorrhoea, rectal gonorrhoea, urethral chlamydia, rectal chlamydia or syphilis these were classified as concurrent infections.

e. If a man had any of the 5 infections listed in (d) on 2 or more separate testing episodes in subsequent visits in the 12 month observation period men were classified as having repeat infections.

### STI testing rates among men in the reminder group compared to men in the concurrent control group

The proportion of men testing at least once during the 12 month observation period for each recommended test - pharyngeal gonorrhoea, rectal gonorrhoea, urethral chlamydia, rectal chlamydia, syphilis and HIV - was significantly higher among men receiving 3 and 6-monthly reminders than among men in the conccurrent control groups with the highest rates of testing among men receiving 3-monthly reminders. The proportion of men who had all of the above tests performed at a given subsequent visit was significantly higher among men receiving 3 and 6-monthly reminders than men in the concurrent control group (*p*<0.001) with the highest complete testing rate (55.7%) among men receiving 3-monthly reminders (Table 2). Complete testing refers to testing for pharyngeal and rectal gonorrhoea, urethral and rectal chlamydia, syphilis and HIV all at the same clinic visit. Compared to men in the concurrent control group, men in the offered reminder group had significantly higher testing rates for all above-mentioned STIs (Table 2).

### STI detection rates among men in the reminder group compared to men in the concurrent control group

Compared to men in the conccurrent control group, men receiving the 3-monthly reminders had a significantly higher detection rate during the 12 month observation period for: rectal gonorrhoea (3.7% vs 1.2%, *p* = 0.001), urethral chlamydia (1.4% vs 3.1%, *p* = 0.027), rectal chlamydia (2.8% vs 6.6%, *p*<0.001) and early syphilis (1.5% vs 3.2%, *p* = 0.025), including early latent syphilis (0.4% vs 1.7%, p = 0.008). No difference in detection of pharyngeal gonorrhoea or HIV was seen. There was a significantly higher proportion of men who had two or more infections detected at separate testing episodes during the 12 month observation period among men receiving 3-monthly reminders compared to men in the concurrent control group (2.6% vs 0.7%, *p* = 0.003). No differences in detection rates were seen between men receiving 6-monthly reminders and men in the concurrent control group. Compared to men in the concurrent control group, men in the offered reminder group only had significantly higher detection rate for early latent syphilis (Table 2). There were no differences in the overall yield of testing when men receiving reminders were compared with men in the concurrent control group (Table 2).

### Factors associated with complete testing in the post intervention period

Multivariate logistic regression showed complete testing - that is, testing for pharyngeal and rectal gonorrhoea, urethral and rectal chlamydia, syphilis and HIV all at the same clinic visit - during the post intervention period was independently associated with: acceptance of reminders (adjusted odds ratio(AOR) = 1.7, 95% confidence interval (CI):1.5–2.1); younger age (AOR = 1.4, 95% CI:1.2–1.6); and a higher number of reported male sexual partners (AOR = 1.6, 95% CI:1.4–1.9) (Table 3).

**Table 3:** Factors associated with complete testing among men in the post intervention group.

	Complete	Incomplete	Crude OR	Adjusted OR
	testing^a^ (n)	testing^a^ (n)	(95% CI)	(95% CI)
**Age group**				
≤30 years	1163	2071	1.54 (1.38–1.71)	1.34 (1.20–1.58)
>30 years	825	2255	1	1
**No. of male partners in past 3 months**				
0–2	712	1923	1	1
≥3	1041	1657	1.70 (1.51–1.91)	1.61 (1.39–1.86)
**Condom use with male partners in past 3 months**				
Never	185	490	1	1
Ever	1470	2589	1.50 (1.26–1.80)	1.13 (0.90–1.42)
**Accepted reminders**				
Yes	494	503	2.12 (1.84–2.44)	1.77 (1.51–2.08)
No	1114	2403	1	1

a Complete testing refers to testing for pharyngeal gonorrhoea, rectal gonorrhoea, urethral chlamydia, rectal chlamydia and serology for both HIV and syphilis at the same visit, **at subsequent visits in the 12 month observation period.**

### Clinic visits, STI testing and detection rates among men in the post intervention group compared to men in the historic control group

The proportion of men who re-attended at least once, or were tested for any infection or received complete testing during the 12 month observation period was greater in the post intervention group than in the historic control group even though only 15% (656/4514) of clinic attenders received 3-monthly reminders and only 7% (301/4514) received 6-monthly reminders.

Similarly a higher proportion of men were diagnosed with rectal gonorrhea (1.7% vs 1.0%, *p* = 0.045), urethral chlamydia (2.1% vs 1.0%, *p* = 0.004) and rectal chlamydia (3.3% vs 1.5%, *p* = 0.001) in the post intervention group during the 12 month observation period when compared with men in the historic control group. Except for rectal chlamydia, no differences in the overall yield of testing were found among men in the post intervention and historic control groups (Table 4).

**Table 4:** Comparison of STI testing and detection rates among men in the post intervention group and men in the historic control group.

	Historic control (n = 1800)		Post intervention (n = 4514)		
	n	%	n	%	*P^b^*
% men who re-attended at least once	1454	80.8	3542	78.5	0.041
No. subsequent visit in the 12 month	2 (1–30)		2 (1–36)		0.203
observation period (median, range) ^a^					
No. (%) of men tested at least once					
for each STI at subsequent visits in					
the 12 month observation period					
Pharyngeal gonorrhoea	495/1454	34.0	1616/3542	45.6	<0.001
Rectal gonorrhea	438/1454	30.1	1486/3542	41.9	<0.001
Urethral chlamydia	535/1454	36.8	1734/3542	49.0	<0.001
Rectal chlamydia	443/1454	30.5	1494/3542	42.2	<0.001
Syphilis	566/1454	38.9	1740/3542	49.1	<0.001
HIV	504/1454	34.7	1620/3542	45.7	<0.001
Complete testing^c^	313/1454	21.5	1250/3542	35.3	<0.001
No. (%) of men diagnosed with an					
infection at least once at subsequent					
visits in the 12 month observation					
period					
Pharyngeal gonorrhoea	11/1454	0.8	49/3542	1.4	0.065
Rectal gonorrhea	14/1454	1.0	61/3542	1.7	0.045
Urethral chlamydia	14/1454	1.0	76/3542	2.1	0.004
Rectal chlamydia	22/1454	1.5	116/3542	3.3	0.001
Early syphilis	30/1454	2.1	62/3542	1.8	0.453
Early latent syphilis	15/1454	1.0	23/3542	0.6	0.158
HIV	10/1454	0.7	28/3542	0.8	0.653
Proportion of all tests positive at					
subsequent visits in the 12 month					
observation period					
Pharyngeal gonorrhoea	11/722	1.5	51/2624	1.9	0.459
Rectal gonorrhea	15/638	2.3	65/2367	2.7	0.582
Urethral chlamydia	16/786	2.0	81/2830	2.9	0.205
Rectal chlamydia	22/646	3.4	133/2369	5.6	0.024
Early syphilis	31/834	3.7	76/2788	2.7	0.138
HIV	10/729	1.4	28/2516	1.1	0.567

Notes:

a. All subsequent visits were within 12 months of the first visit after entry into the study.

b. P value from chi-squared test calculating the difference in proportion of testing and detection rates between postintervention and historic groups

c. Complete testing refers to a swab for pharyngeal gonorrhoea, rectal gonorrhoea, urethral chlamydia, rectal chlamydia and serology for both HIV and syphilis at the same visit, at subsequent visits in the 12 month observation period.

## Discussion

In this study we have demonstrated that a fully automated reminder service incorporating CASI together with reminder text and email messages was associated with increased testing and detection of several clinically important STIs among MSM: rectal gonorrhoea, urethral and rectal chlamydia, and early, latent syphilis. There was also a significant association with increased detection of repeat infections. We believe this is the first published study that has demonstrated the impact of such a fully automated system in improving STI detection in any population.

A strength of this study is the general consistency of the results using a number of different control groups. There are however some limitations. Firstly this was an retrospective observational study and therefore is subject to potential biases that may arise if there are systematic differences between our comparison groups. To improve the validity of our findings we used a number of control groups. We used a concurrent control group from the post-intervention period which consisted of men who were not offered the reminders. There were a number of differences between those who accepted the reminders and those who were not offered them, with the former being younger, reporting more male sexual partners but using condoms more frequently. It is possible that younger men were more likely to accept the reminders because they were more technology savvy and thus more accepting of the use of new technology. Furthermore, men who accepted reminders may have had a higher self-perceived risk and greater awareness of their sexual health. However, the reminders were still found to be independently associated with a higher rate of testing after adjusting for these differences. We also analyzed results for all men in the post intervention period compared to men in a historic control period – preceding the reminders – and found results that were to some extent consistent with the analysis involving the concurrent control group. The magnitude of the effect seen using the historic control period was less than the concurrent control period but this would be expected given that only a relatively small proportion of those who were offered the reminders accepted them. Another study limitation was that we used culture to test for gonorrhoea. Culture is relatively insensitive for phayngeal and rectal gonorrhoea therefore overall detection rates in all groups could be higher if nucleic acid amplification testing was used instead [23].

We found that men who received the most frequent reminders were those who had the highest number of clinic visits as well as the highest rates of testing with the greatest detection of STIs. Detection rates among men receiving 3-monthly reminders, were for several infections, double that of men who were not offered reminders. While differences in detection rates may to some extent reflect differences in underlying prevalences of infection between men receiving reminders and men in the two control groups, the absence of substantial differences in the yield of testing between groups suggests that differences in detection were not because of major differences in underlying prevalence rates. That testing rates were not as high in the overall clinic population of MSM following the intervention was not surprising given that only 22% of these men were receiving the reminders. The offer of the reminders was framed in terms of the syphilis epidemic and regular blood testing for syphilis. It is possible uptake could be increased by including other STIs and by improving the way in which the reminders are promoted. Given the effect on testing and detection was lower among men offered the reminders than among those receiving 3-monthly reminders greater uptake would be expected to improve overall outcomes.

In a systematic review of controlled studies of clinic-based interventions aimed at increasing bacterial STI screening among MSM, two studies demonstrated increased detection of early [18], [19], latent syphilis but no studies have demonstrated improved detection of either chlamydia or gonorrhoea [17]. Only one of the bacterial STIs in this study was not shown to have improved detection: pharyngeal gonorrhoea. Limited available data suggest that gonorrhoea infection of the pharynx is a relatively transient infection, that is, self limiting in the absence of treatment [24], [25]. Presumably, even more frequent screening would be required to significantly improve detection of gonorrhoea in the pharynx.

A notable aspect of our intervention is that the system was fully automated: MSM were identified by a computer, were offered reminders by the computer, and reminders were sent directly from the clinic database to email addresses or mobile telephones at the chosen frequency without any clinic staff supporting or overseeing the system. Given the minimal cost of emails and text messages and the significant increase in detection rates for several infections, we believe the cost effectiveness of this intervention is likely to be high. Text message reminders have been shown to improve STI testing among women and heteroxual men [26].

The findings in this study support the principle of frequent screening of high risk MSM to improve detection of bacterial STIs, as recommended in guidelines [12,13. Wider application of the automated reminder system employed in this study, or similar ones, together with efforts to encourage high uptake could enhance the control of STIs among MSM.
